# Computational Design of a Tetrapericyclic Cycloaddition and the Nature of Potential Energy Surfaces with Multiple Bifurcations

**DOI:** 10.1021/jacs.2c12871

**Published:** 2023-02-09

**Authors:** Ana Martin-Somer, Xiao-Song Xue, Cooper S. Jamieson, Yike Zou, K.N. Houk

**Affiliations:** † Departamento de Química Física Aplicada, Facultad de Ciencias, Módulo 13, Universidad Autónoma de Madrid, Campus de Excelencia UAM-CSIC Cantoblanco, 28049 Madrid, Spain; ‡ Department of Chemistry and Biochemistry, University of California, Los Angeles, Los Angeles, California 90095, United States

## Abstract

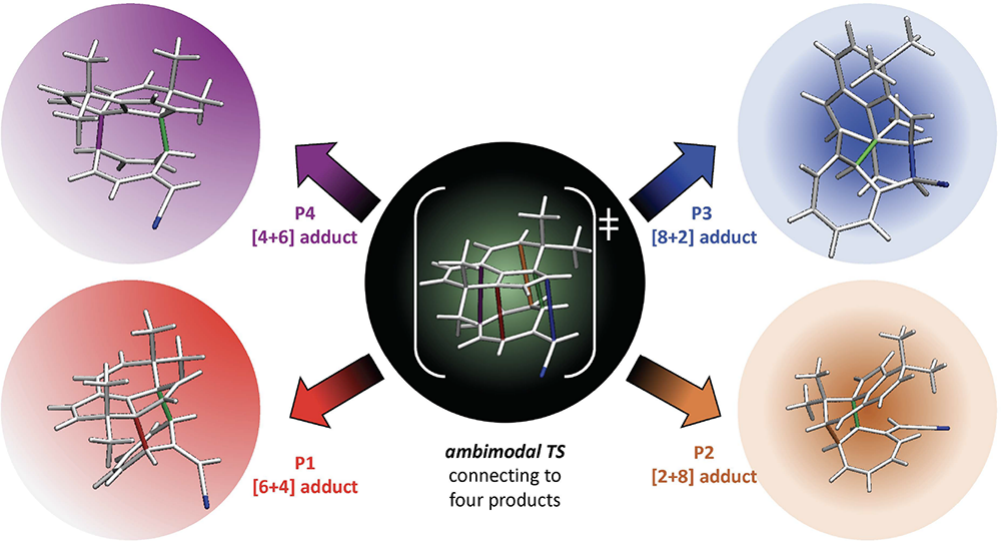

An ambimodal transition state (TS) that leads to formation of four different pericyclic reaction products ([4 + 6]-, [2 + 8]-, [8 + 2]-, and [6 + 4]-cycloadducts) without any intervening minima has been designed and explored with DFT computations and quasiclassical molecular dynamics. Direct dynamics simulations propagated from the ambimodal TS show the evolution of trajectories to give the four cycloadducts. The topography of the PES is a key factor in product selectivity. A good correlation is observed between geometrical resemblance of the products to the ambimodal TS (measured by the RMSD) and the ratio of products formed in the dynamics simulations.

## Introduction

Post-transition state bifurcations (PTSB) have been observed in many reactions as evidenced by the increasing number of publications related to this subject.^1^ Starting with the first ambimodal bispericyclic cycloaddition reported by Caramella et al.^2^ in 2002, related transition states with bifurcations have been reported in cycloadditions involved in organic synthesis, biosynthesis, organocatalysis, organometallic, and enzymatic reactions, as previously reviewed by Houk et al.,^3^ Carpenter and Rehbein,^4^ and Tantillo and Hare.^1^ In fact, one of the earliest reports of a PTSB was in the electrocyclic ring opening of cyclopropylidine to form allene, studied by Ruedenberg et al. in the 1990s.^5^ Many of these ambimodal transition states (TSs) involve a single post-transition state bifurcation. That is, one transition state leads to a reaction coordinate that bifurcates after crossing the TS region into two paths to different reaction products without an intervening minimum. These reactions have a relatively well-defined valley-ridge inflection region where the bifurcation occurs and are of the “two transition state, no intermediate” type, as defined by Houk and co-workers*.*^6^

Our group recently reported the first example of ambimodal tripericyclic TS where the reaction coordinate splits into three different paths leading to three different cycloaddition products as a result of sequential bifurcations.^7^ Several other reactions with ambimodal transition states and formation of three products have been reported.^8^ Examples of multiple sequential TSs have been shown previously in the literature by Hong and Tantillo.^9^

**Figure 1:**
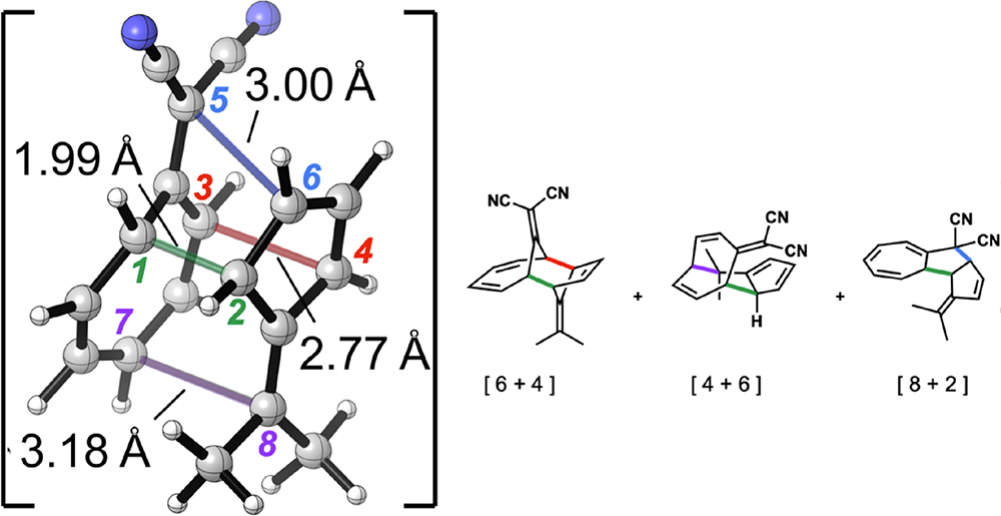
Ambimodal tripericyclic transition state and the three products that are formed.^7^

Figure 1 shows an ambimodal TS that leads to the three adducts shown.^7^ The bond between the green atoms, 1 and 2, is the primary interaction and leads to a bond in all three products. The other colored atoms involve what we call conditional primary interactions; red 3 and 4 form a bond in the [6 + 4] adduct, purple 7 and 8 form a bond in [4 + 6], while blue 5 and 6 give a bond in [8 + 2].

We have shown how the surface involves a bifurcation after the TS, and each pathway leads to another transition state and bifurcation before a product is reached.

We now report the design of a reaction featuring a tetrapericyclic TS, which is one transition state that leads to four different cycloaddition products resulting from multiple bifurcations. In order to design such a tetrapericyclic ambimodal TS, we needed to satisfy several requirements: (i) favorable orbital interactions between addends, leading to five partial bonds in the TS (one primary and four conditional primary) that can lead to four products; (ii) conditional bonding interactions that should be within 0.4 Å of each other in the first TS in order to give some of each of the four products;^10^ (iii) the ambimodal TS energy should be lower than that of competing reactions; (iv) the reaction should be feasible in the lab (Δ*G*^‡^ < 30 kcal/mol); and (v) the products should be stable and observable. We were able to achieve the first four criteria, at least theoretically, but all four products may not be observable experimentally.

**Figure 2:**
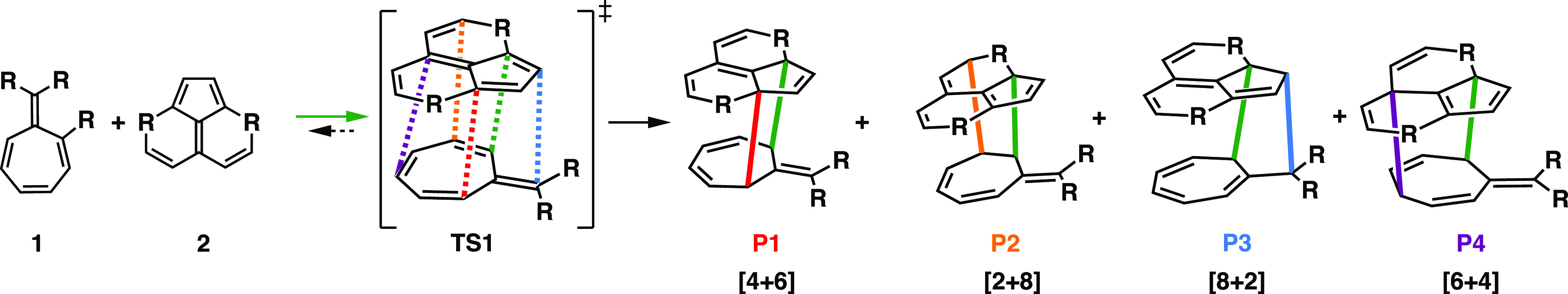
Ambimodal transition state, **TS1**, formed from the reaction of **1** and **2** and the four possible products **P1**, **P2**, **P3**, and **P4** corresponding to [4 + 6]-, [2 + 8]-, [8 + 2]-, and [6 + 4]-cycloadducts, respectively.

Because of our experience with cycloadditions of pentafulvene and heptafulvene derivatives and the involvement of these cyclic polyenes in many ambimodal cycloadditions, we explored the extension of the conjugation in the fulvene to open up possible formation of new products.^11−14^ Both types of fulvenes are polarized and reactive due to the tendency of the odd-membered rings to assume aromatic character. By adding a 6-vinyl group to fulvene, we created an 8π electron system; we made the system symmetrical by adding two 6-vinyl groups (10π electron system). We also made these fulvene derivatives cyclic to eliminate conformational freedom. The general systems, **1** and **2**, shown in Figure 2, were explored to find a system that can give a tetrapericyclic reaction.

## Results and Discussion

Figure 2 shows the general geometry of an ambimodal transition state, **TS1**, that can be formed from the reaction of **1** and **2** and can lead, in principle, to four different pericyclic cycloaddition products.

The alignment of addends shown has five partially formed σ-bonds: B0, B1, B2, B3, and B4. B0 is the primary bonding interaction shown in green and present in all possible adducts, while B1–B4 are conditional primary interactions, only one of which can form in a single trajectory to lead to one of the products, **P1–P4**.

**Figure 3:**
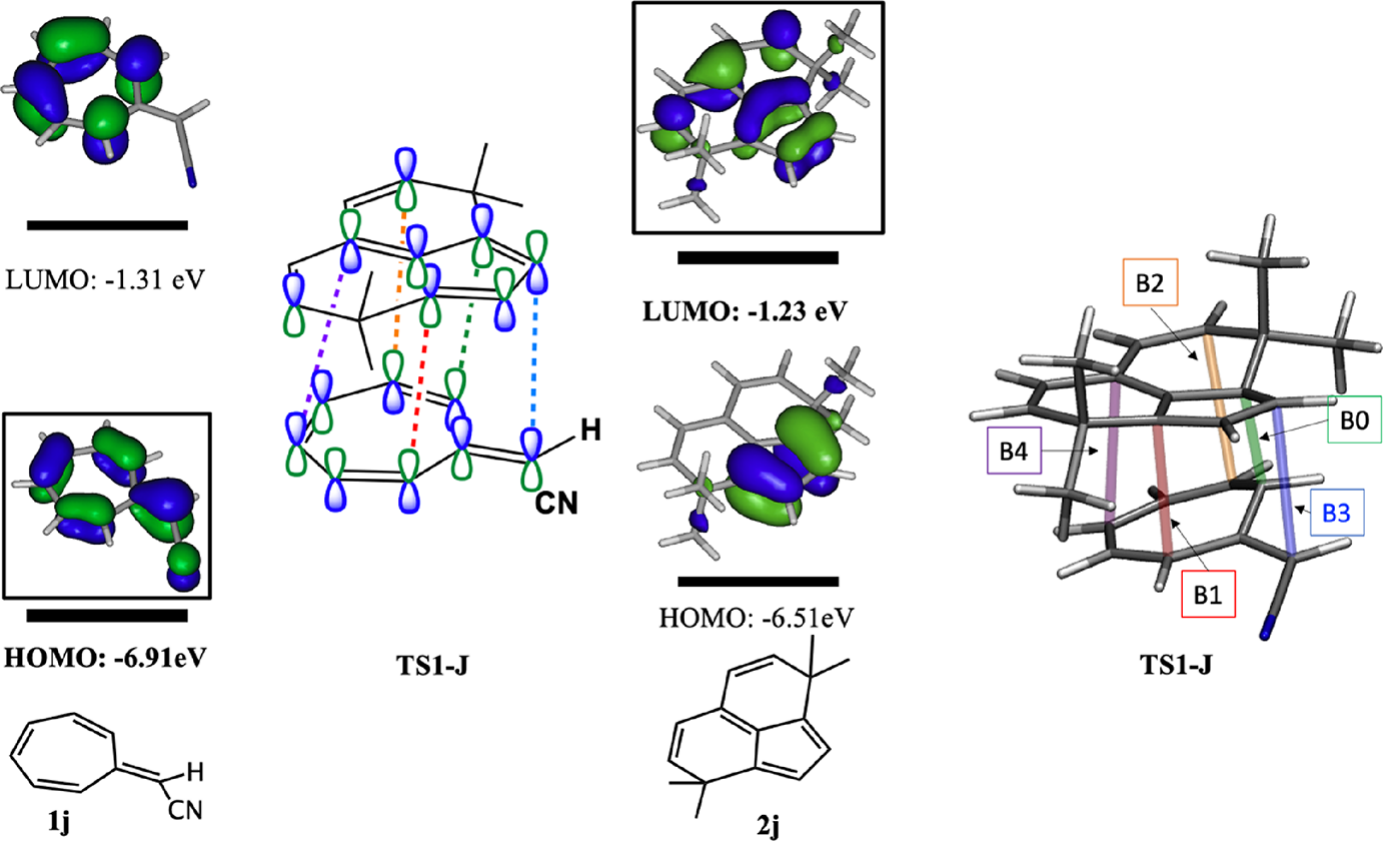
HOMO and LUMO of 8-cyanoheptafulvene (**1j**) and a tricyclic 6,6-divinyl pentafulvene derivative (**2j**). The interactions leading to potential ambimodal tetrapericyclic reactions are shown in **TS1-J**. Right: Optimized structure for **TS1-J**.

The HOMO and LUMO of cyanoheptafulvene (**1j**) and a tricyclic 6,6-divinyl pentafulvene derivative (**2j**) are shown in Figure 3. These are the reactants that we eventually found that can form a tetrapericyclic transition state that can lead to four products. The orbitals shown have the appropriate symmetry to give four different products: [4 + 6]-, [2 + 8]-, [8 + 2]-, and [6 + 4]-cycloadducts from five partial bonds in the transition state. Note how the signs of the HOMO coefficients of 8-cyanoheptafulvene (**1j**) match with those of the divinylpentafulvene (**2j**) LUMO. The interaction between reactants is high because of these orbital interactions, although the energy gap is a little larger than for the other HOMO–LUMO interaction (5.7 vs 5.2 eV). The latter strongly stabilizes the primary interactions leading to the product **P4**, which we later show is dynamically preferred for this reaction. Because of the unsymmetrical CN substitutions, there are four stereoisomeric adducts possible, which have the stereochemistry of the cyano group reversed.

We explored the 12 combinations of substituted heptafulvenes (**1**) and tricyclic divinylpentafulvenes (**2**) shown in Table 1. Frontier molecular orbitals for the heptafulvene and pentafulvene derivatives are shown in Figures S1 and S2, respectively. Table 1 shows the free energy of each of the 12 TSs and the energies of the four possible products, **P1–P4**, that can be formed from **TS1**.

**Table 1:**
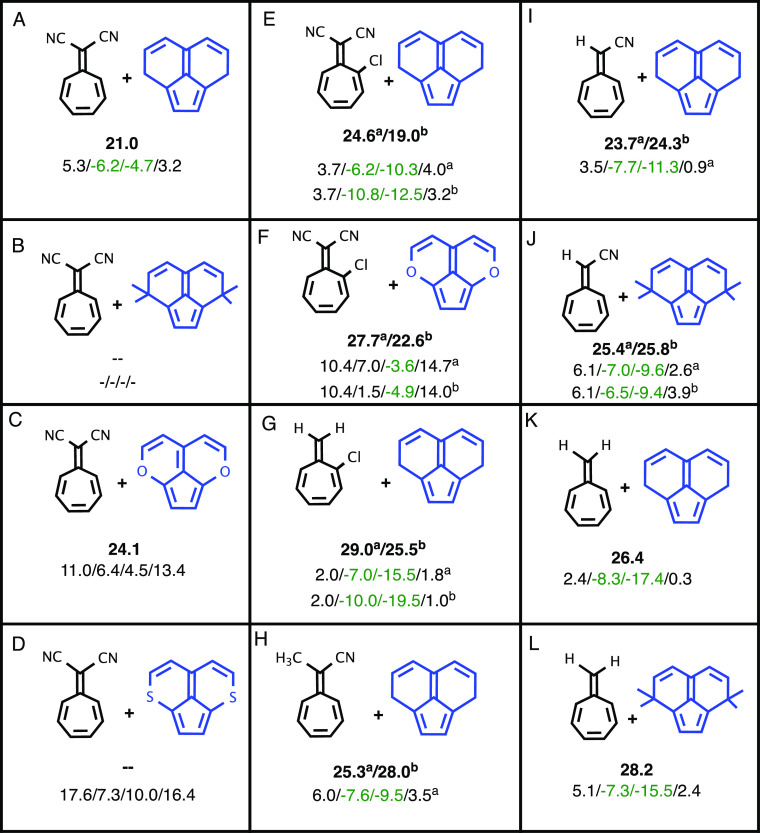
Cycloaddition Reactions Studied: TS1 Gibbs Free Energies (Bold Number) and the Corresponding Product Energies (P1/P2/P3/P4) Calculated with M06-2X/6-31G(d) Are Shown for 12 Possible Reactions between **1** and **2**

All energies are in kcal/mol. The letters a and b are used when two **TS1** orientations are possible for the non-symmetrical cases (E–J). For entries B and D, it was not possible to optimize **TS1**. For entry B, we did not compute product energies either.

Entries A–D in Table 1 compare reactions of dicyanoheptafulvene with different tricyclic divinylpentafulvene derivatives. For entry B, it was not possible to optimize **TS1** and we did not optimize the products. For the eight other reactions, only two products are predicted to be exoergic, the [2 + 8] and [8 + 2] adducts, **P2** and **P3**. Unfortunately, we were unable to achieve one of our criteria, the ability to isolate and characterize all four products due to thermodynamic stability. Nevertheless, we did achieve the other four criteria with system J.

**Figure 4:**
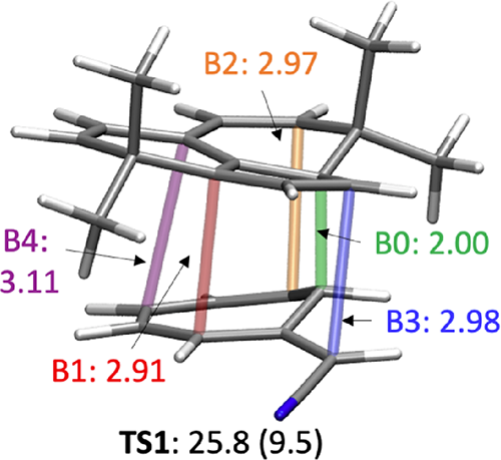
Ambimodal tetrapericyclic **TSI-J**. Bond lengths in Å. Gibbs free and (potential) energies in kcal/mol.

For the thio derivative (D), we could not find **TS1**, and all four products are less stable than the reactants. Entries E–G show that adding a chlorine atom to the heptafulvene derivative **1** stabilizes the products. Since **1** is not symmetric, there are two possible orientations for **TS1** (a and b) with different energies. When B0 is formed between the heptafulvene carbon that is not substituted by the Cl atom (**TS1^b^**), **TS1** is more stable (E^b^ vs A, F^b^ vs C, and G^b^ vs K). Reactions with heptafulvene (K and L) and cyanoheptafulvene (I and J) also yield stable products (Δ*G* < 0) with **TS1** energies slightly higher than for reactions with dicyanoheptafulvene (entry A). As shown in Figure S3, we added two methyl groups to each methylene carbon (entries B, J, and L). The geometry of **TS1-J^b^** is displayed in Figure 4. We will refer to it as **TS1-J**.

**TS1-J** features five partially formed σ-bonds (B0, B1, B2, B3, and B4) and is highly asynchronous. B0 is the primary interaction with a length of 2 Å, considerably shorter than the conditional primary interactions B1, B2, B3, and B4 that are 2.9–3.1 Å (Table 2 and Figure 4). This is a general feature in ambimodal TSs for cycloadditions,^7,8,10^ that is, the primary interaction is about 2 Å, and the conditional primary interactions are about 3 Å. We have previously described a bond length criterion to predict the outcome of dynamics simulations that started from an ambimodal TS: when the difference between two bond lengths is 0.40 Å or greater, the percentage of the major product obtained in the dynamics simulations is larger than 98%.^10^ We also found a roughly linear correlation between conditional bond lengths and product ratios. Since B1–B4 bonds are in a 0.2 Å range, we expect these four bonds to compete dynamically to form four products in the dynamics simulations. This is tested and verified later in this paper by molecular dynamics simulations.

**Table 2:** Key Bond Lengths and Potential and Gibbs Free Energies for the Ambimodal TS, **TS1**, and the Three TS Interconverting Products **TS2**, **TS3**, and **TS4** (Interconverting **P1**–**P4**, **P2**–**P4**, and **P3**–**P4**, Respectively) for Reaction J^b^

	B0 [Å]	B1 [Å]	B2 [Å]	B3 [Å]	B4 [Å]	Δ*E*^‡^ [kcal/mol]	Δ*G*^‡^ [kcal/mol]
**TS1-J**	2.00	2.91	2.97	2.98	3.11	9.5	25.8
**TS2-J**	1.64	2.53	3.02	3.06	3.00	5.4	23.7
**TS3-J**	1.61	3.16	2.53	3.16	2.93	5.5	23.2
**TS4-J**	1.62	3.02	3.02	2.44	3.16	7.8	25.6

We also studied all the possible dimerization reactions of **2J** to ensure that there were no competing reactions (Figure S4). All the energy barriers are higher than the barrier for the ambimodal reaction.

**Figure 5:**
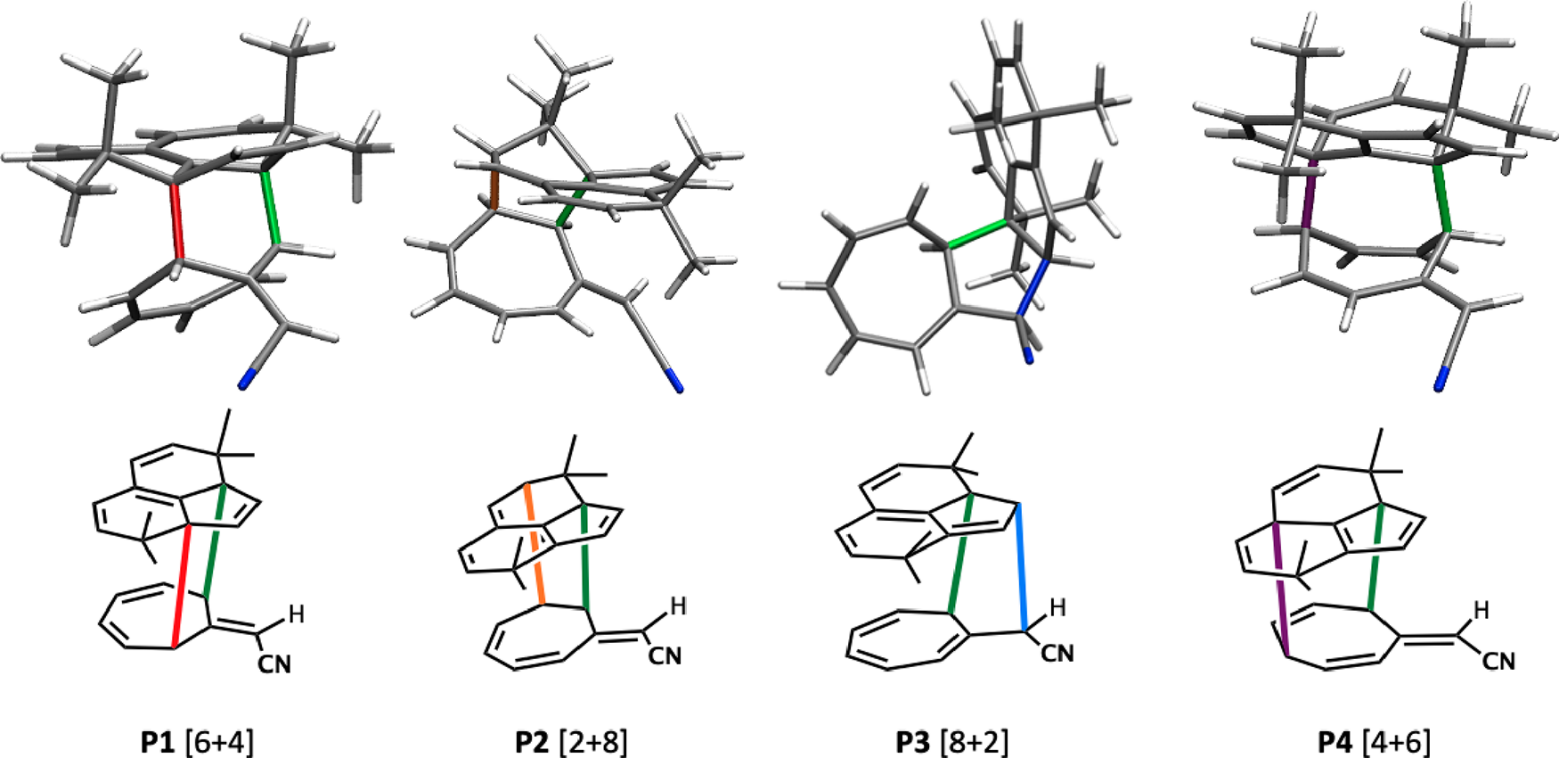
Optimized structures of **P1-P4** formed from **TS1-J**. The new CC bonds formed are highlighted in colors.

The structures of the four products are shown in Figure 5.

**Figure 6:**
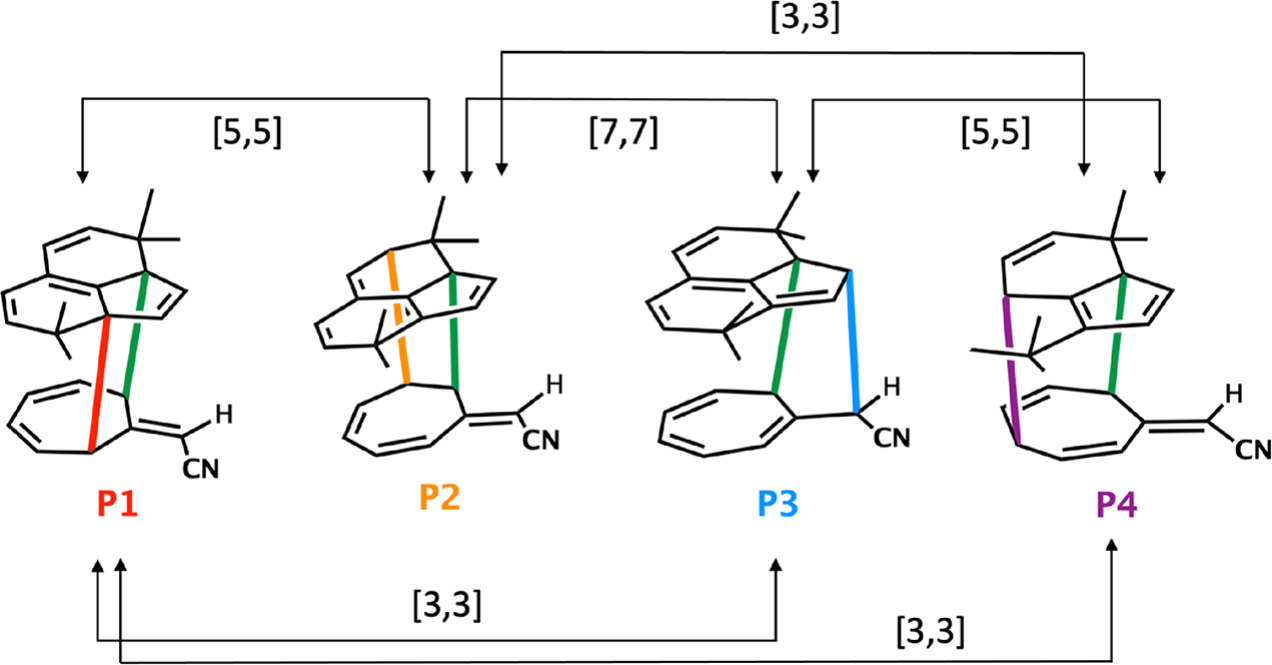
Potential possible product interconversions.

We also located transition states interconverting pairs of products. Figure 6 shows diagrammatically that six interconversion TSs are feasible, three [3,3], two [5,5], and one [7,7] sigmatropic shifts, and all of these are all allowed, supra-supra sigmatropic shifts involving 4n + 2 electrons.

**Figure 7:**
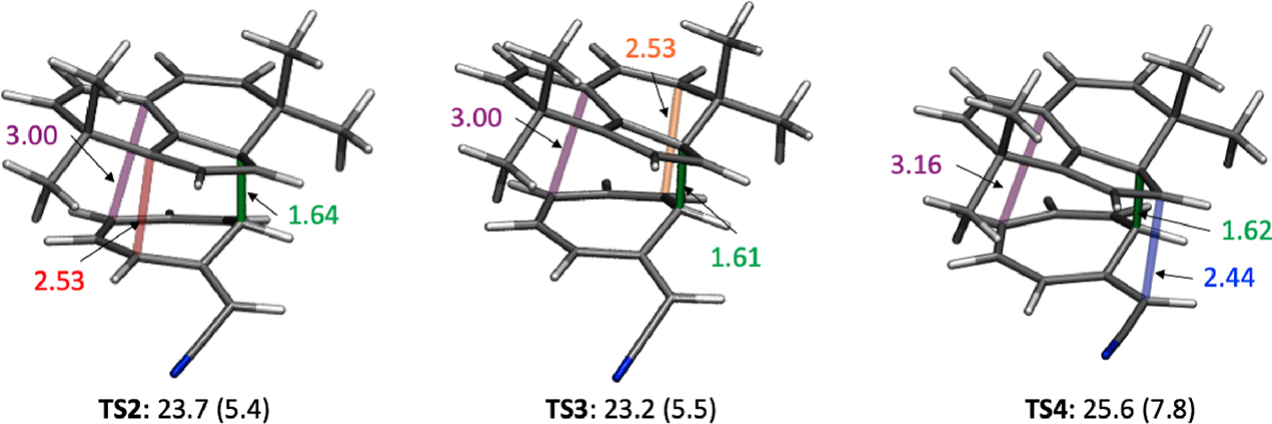
**TS2–TS4** that interconvert products. All have B0 = 1.61–1.64 Å already formed. **TS2** interconverts **P1** and **P4**; **TS3** interconverts **P2** and **P4**; **TS4** interconverts **P3** and **P4**. Bond lengths in Å and Gibbs free energy and potential energy (within parentheses) in kcal/mol are also shown.

We found three of these interconverting TSs, although there could in principle be six. For example, **P1** can interconvert with **P2**, **P3**, and **P4**, while **P2** can interconvert with **P1**, **P3**, and **P4**, etc. The structures of the post-ambimodal TS transition structures (**TS2**–**TS4**) that were located are shown in Figure 7. **TS2**–**TS4** all connect **P4** with one of the other products by [3,3], [3,3], and [5,5] sigmatropoic shifts, respectively.

**Figure 8:**
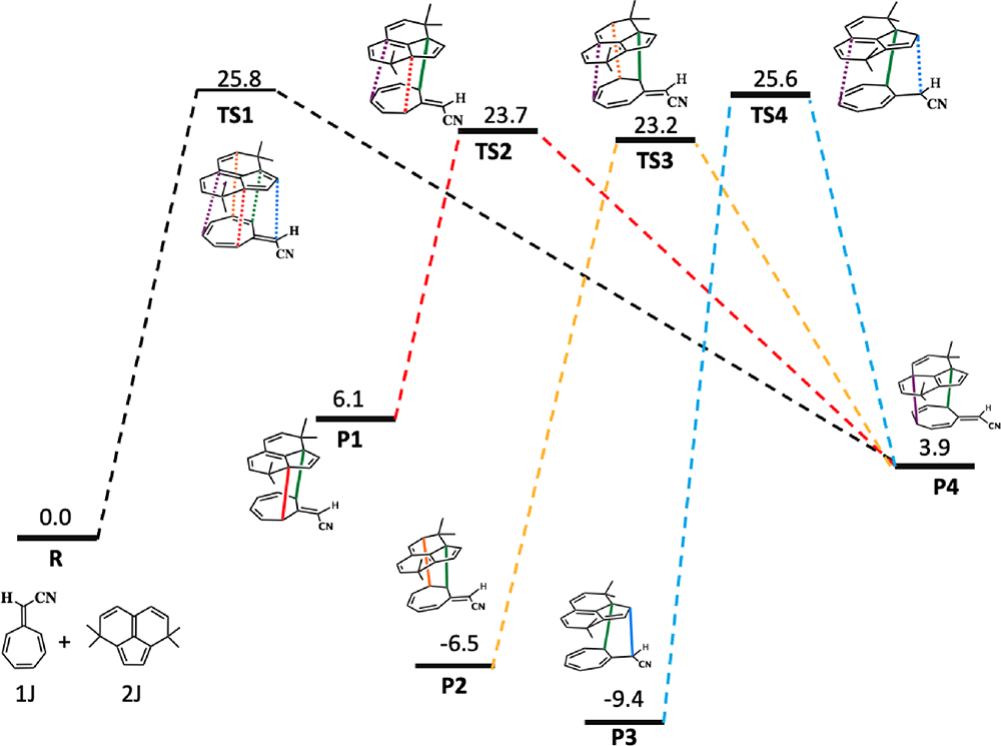
Stationary points located for the reaction of **2J** with **1J**. Free energies are given in kcal/mol. Bold lines correspond to already formed bonds, while dashed lines are forming and breaking bonds.

All the stationary points located for the reaction of 1j with 2j are shown in Figure 8. The dashed lines connect each TS with the two stationary points linked to the TS by the intrinsic reaction coordinate (IRC) calculations.

### IRC Calculations

A PES with PTSB is characterized by the presence of two consecutive TS, **TS1** and **TS2**, without an intervening minimum. Thus, the absence of an intermediate is a good indication of the presence of a bifurcation.^15,16^

We calculated IRCs for selected **TS1**s. The IRC curves are shown in Figure S5. If the IRC calculation stops at an intermediate (stepwise reaction), then the TS is not ambimodal and was, therefore, discarded. Such is the case for reactions A and E^b^. The rest of the reactions are concerted asynchronous processes; there are no intermediates between TSs and products. The IRC leads to one of the possible products.

**Figure 9:**
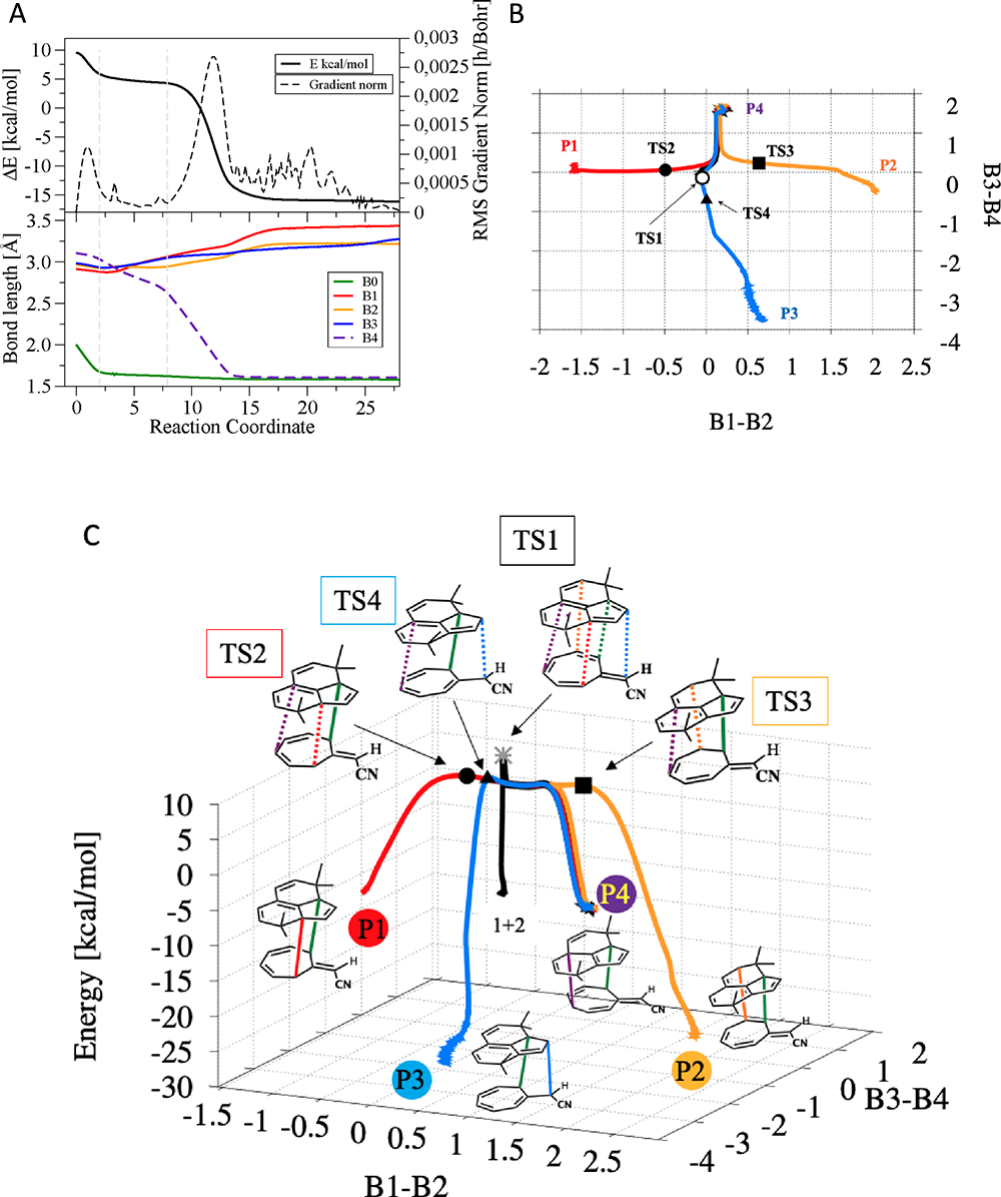
IRCs from **TS1–TS4**: (A) IRC calculation for ambimodal **TS1** in the gas phase. Only the part corresponding to TS to product B4 formation is shown. In the bottom part of the figure are displayed the bond lengths of the five partially formed σ-bonds (B0, B1, B2, B3, and B4). (B) IRC from **TS1–4** plotted on the reduced dimensionality surface obtained by plotting in the *x* axis the value of B1–B2 and in the *y* axis B3–B4 bond lengths. The empty circle, filled circle, triangle, and square mark the coordinates for **TS1**, **TS2**, **TS3**, and **TS4** respectively. (C) Same as B, but adding the potential energy in the *z* axis. The structure of products and TS are also shown. Dashed lines mark partially formed/broken bonds. More perspectives of this plot can be found in Figures S7 and S8.

Figure 9A displays the computed IRC (only the part leading to products, **P4** in this case) for reaction J^b^ along with B0–B4 bond lengths. The IRC computed with implicit solvent is shown in Figure S6.

Figure 9A shows three regions, marked with the gray dashed vertical lines. In the first region, the energy decreases with a steep slope while the primary bond, B0, (black curve) is formed. In the second region, the IRC curve is flat, but the gradient (gray curve on the top graph) does not quite reach a zero value; the structure is not a potential energy intermediate but is what we call an entropic intermediate. Such flat regions are generally encountered along bifurcating pathways.^17,18^ The potential energy does not vary much, but there are important structural rearrangements in both reactants. There is a slight initial decrease in the bond length of the four bonds (B1–B4), while B4 shortens by 0.4 Å. The lengths of B1–B3 increase. In the third region, the potential energy decreases sharply again, and a second bond is formed, B4, yielding **P4**. B1, B2, and B3 steadily increase in the third region till their final values around 3.3 Å. The IRC algorithm leads to one of the possible products, **P4**, in all the cases listed in Table 1, except for G^b^ that leads to **P1**.

### Product Interconversion Transition States: **TS2**, **TS3**, and **TS4**

**TS1** leads to four products, **P1**, **P2**, **P3** and **P4**, via the subsequent **TS2**–**TS4**. As noted in Figure 6, there are six possible TSs interconverting those products. We located three: **TS2**, **TS3**, and **TS4** connecting **P1**–**P4**, **P2**–**P4**, and **P3**–**P4**, respectively. The three TSs are lower in energy (potential and Gibbs) than **TS1** (Table 2 and Figure 7).

In Figure 9B,C, IRCs for **TS1–4** are drawn on the 2D surface used to map the evolution of relevant bond lengths for the reaction: B1 to B4. We have reduced the dimensionality by plotting the value of B1–B2 on the *x* axis and B3–B4 on the *y* axis. **TS1** IRC goes from reactants (**1** + **2**) to **P4**. The three IRC converge with **TS1**-IRC in the flat part leading to **P4**. Furthermore, **TS2** and **TS3** IRCs are orthogonal to **TS1**-IRC, another common feature of bifurcating PES. Also, it is worth noting that **P3**, **TS4**, **TS1**, and **P4** form a (an almost) straight line, however the PES looks much steeper going toward **P4** than toward **TS4**/**P3**. Indeed, **TS4** is the closest in energy to **TS1** (see Figures S7 and S8).

### Dynamics

Different attempts have been made to relate geometrical features in the TS, such as bond lengths, with product ratios observed in ambimodal reactions.^10,19,20^ Singleton et al. refer to this as the “static factor”.^21^ It has also become clear that dynamic effects, which can only be taken into account by means of dynamics simulations, can play an important role in product selectivity.^4,9,15,22,23^ For example, Hong and Tantillo showed a terpene synthesis with multiple sequential post-transition state bifurcations leading to many different products. However, only two of those products are observed in a significant amount as the outcome of trajectory simulations. They attributed this phenomenon to dynamic effects “the route to the transition-state region influences the route away from it”.^9^ This phenomenon was called by Carpenter “dynamical matching” where *“*the momentum direction associated with an incoming trajectory initiated at a high energy saddle point determines to a considerable extent the outcome of the reaction”.^24^ Singleton et al. referred to it as the “dynamic factor”.^25^ We have performed dynamics simulations and describe a new way to predict product ratios.

We performed quasiclassical direct dynamics calculations^26^ starting from **TS1** in order to obtain about 100 trajectories each for reactions A, E^a^, H^a^, J^a^, and J^b^. The results are shown in Table S1. In terms of general reactivity, there is a clear difference between reactions A and E^b^ and reactions H^a^, J^a^, and J^b^. It is important to recall that reaction A and E^b^ are stepwise, i.e., before the final products, there is an intermediate where bond B0 has already been formed. This is reflected in the fact that the number of reactive trajectories is much lower, 40 and 24%, respectively, compared to more than 90% of reactive trajectories in the other cases. For reaction A, 13% of trajectories recross the TS region, while 47% form the intermediate. In the case of E^b^, only 3% of the trajectories recross, similar to the other reactions, while most of the trajectories, 73%, stop at the intermediate. This is easily explained by the difference in potential energy between **TS1** and the intermediate in these reactions: 3.6 and 5.2 kcal/mol for A and B^b^, respectively.

Focusing on the product distributions predicted by dynamics, these results are similar for all of the reactions considered, except for reaction J^b^. In most cases, only three of the four expected products are observed in the dynamics simulations. The product distribution is also quite similar (% over reactive trajectories): **P4** is the major product, formed in more than half of the reactive trajectories. The next one is **P2** followed by **P1** observed in about 10% of the reactive trajectories. Interestingly, **P3** is not formed in any of the trajectories, even though it is the most stable of the four products.

In the case of J^b^, the product distribution is different from the others, and all four products are formed. **P4** is the major product, but the percentage of trajectories yielding **P1** is larger than those yielding **P2** (17 vs 11%). Furthermore, 3% of the trajectories yield **P3**. Therefore, four products are observed and it can be confirmed as a tetrapericyclic reaction, with **TS1-J** leading to four products, [4 + 6]-, [2 + 8]-, [8 + 2]-, and [6 + 4]-cycloadducts, without intervening minima.

**Figure 10:**
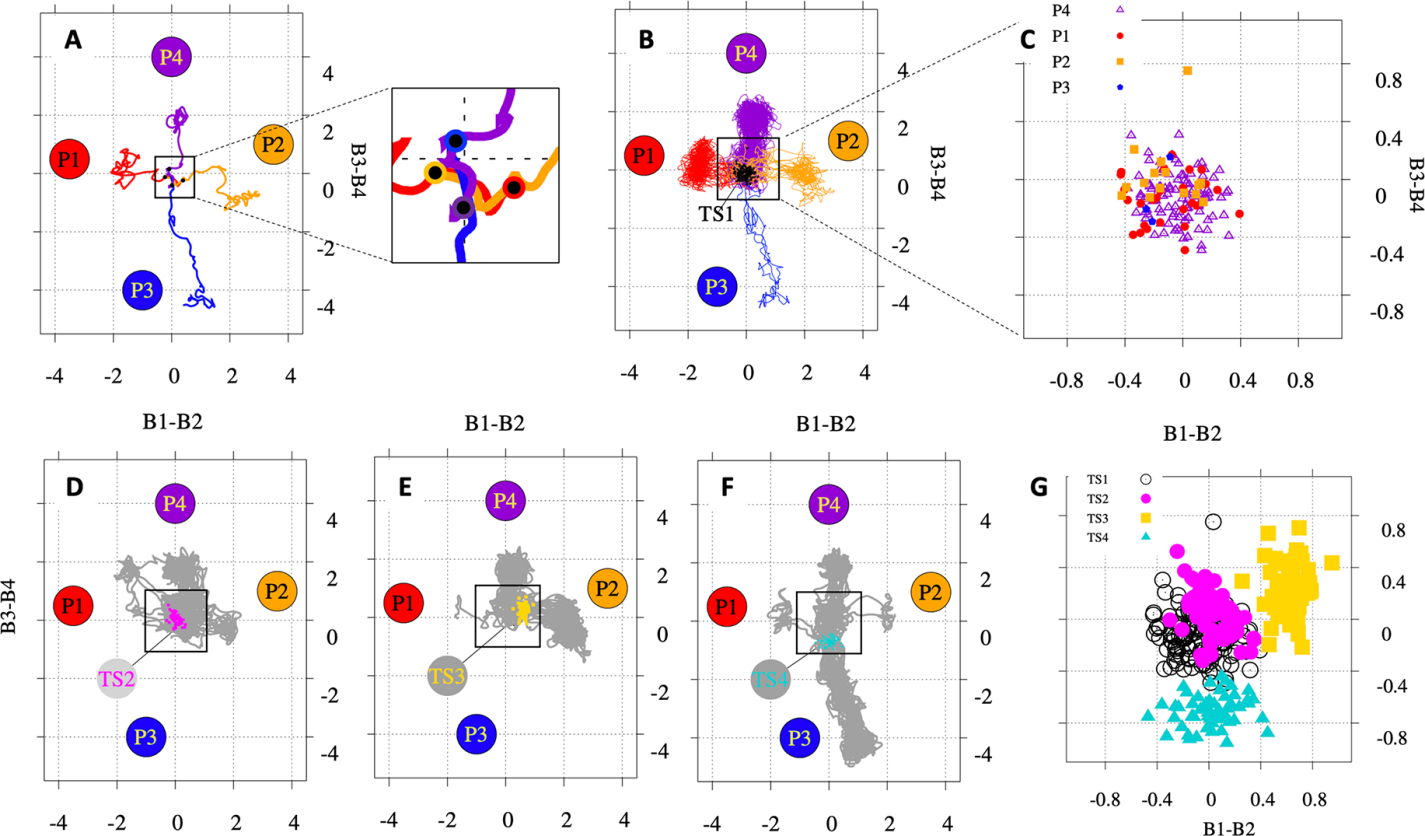
Results of dynamics simulations. Plots of trajectories on the reduced dimensionality surface obtained by plotting in the *x* axis the value of B1–B2 and in the *y* axis B3–B4 bond lengths. Panels (A–C) correspond to trajectories that propagated from ambimodal tetrapericyclic transition structure **TS1**. (A) Four selected trajectories yielding **P1** (red), **P2** (orange), **P3** (blue), and **P4** (violet) cycloadducts. (B) Ensemble of 152 trajectories. The black circles mark the initial geometries for each trajectory. (C) Initial geometries (*x* = B1–B2 and *y* = B3–B4 at *t* = 0 fs) in the sampled ensemble of trajectories that propagated from **TS1** colored by the final product reached **P1** (red circle), **P2** (orange square), **P3** (blue pentagon), and **P4** (violet triangle). Panels (D–F) correspond to trajectories propagating from product interconverting TS, **TS2** (linking **P1**–**P4**), **TS3** (**P2**–**P4**), and **TS4** (**P3**–**P4**). The circles mark the initial geometries for each ensemble. The black line marks the IRC from each interconverting TS. (F) Initial geometries for all the sampled trajectories: **TS1** (black), **TS2** (magenta), **TS3** (gold), and **TS4** (turquoise). All calculations are at the M06-2X/6-31G(d) level of theory. Movies are available in the Supporting Information.

We have analyzed J^b^ in more detail. The top part of Figure 10 shows trajectories that propagated from **TS1** on the reduced dimensionality 2D surface. Panel A shows four representative trajectories, each leading to one of the four products. The red trajectory leading to **P1** crosses all the other three trajectories going almost on top of the orange one (leading to **P2**) for the first part of the trajectory. However, the trajectory keeps its original movement toward the left, yielding **P1**. A similar behavior is observed for the other three trajectories. This is an illustration of the “dynamical-matching” phenomenon referred to previously.

Panel B shows 152 trajectories that propagated from **TS1-J**. The black dots mark the initial geometries (*x* = B1–B2 and *y* = B3–B4 at *t* = 0 fs) for the sampled ensemble. If we color these points as a function of the final product reached by the trajectory (panel C), then we observe that there is no apparent correlation between initial geometries and the final outcome of the trajectory.

The bottom part of Figure 10 shows trajectories that started at **TS2**, **TS3**, and **TS4**. The results for product distribution are summarized in Table S2. As expected, most of the trajectories show the interconversion between the expected products, i.e., the IRC connected products. Nevertheless, some of the trajectories show interconversion between other products, for example, **P2**–**P4** or **P1**–**P2** starting from **TS2** (IRC links **P1** with **P4**). Panel G shows the initial conditions sampled for the trajectories that propagated from each of the four TSs. There is a high overlap between **TS1** and **TS2** initial coordinates, while **TS3** and **TS4** do not overlap with any other.

### Correlation between Geometries and Dynamics Product Distribution

**Figure 11:**
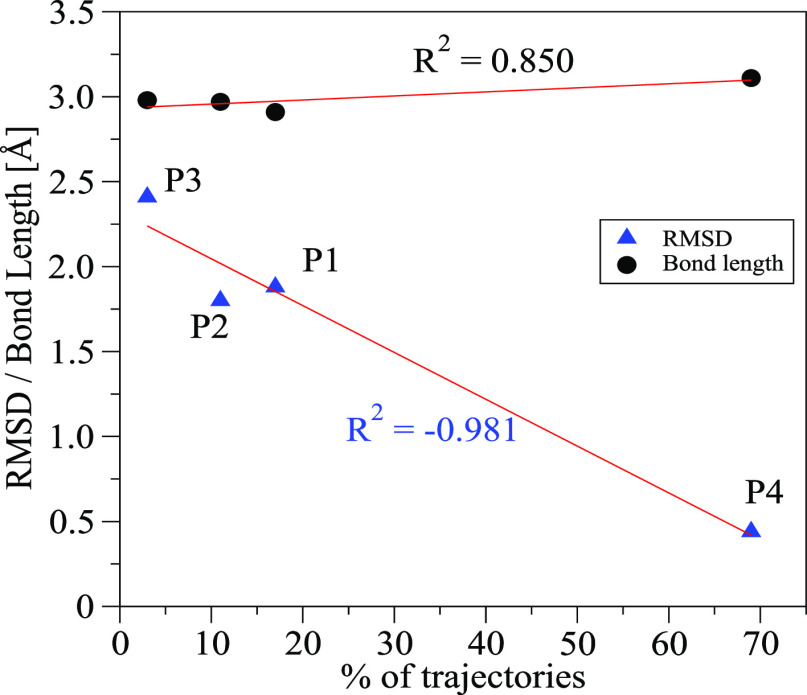
Correlation between RMSD of each product (blue triangles) and bond length on the ambimodal TS (black circles) of the four products and the % of trajectories propagated from **TS1-J** yielding that product. The values can be found in Table S3.

As noted earlier, we previously published a correlation between the bond lengths of the conditional primary interaction in the ambimodal TS and the % of trajectories leading to the corresponding product. Figure 11 shows the correlation between B1–B4 bond lengths at **TS1** and the product distribution. The correlation coefficient is 0.85, but there is not much change in the conditional bond length. One remarkable thing is the fact that the main product from the dynamics, **P4**, is the one with the largest bond length at **TS1** (3.11 Å), exactly the opposite from expectation.

We explored a different relationship and compared the product distribution with the root-mean-square deviation (RMSD) of product geometry from the **TS1** geometry. In this case (also plotted in Figure 11), the correlation coefficient is much higher, 0.981, and we see that the product structure closest to that of **TS1**, **P4** (RMSD = 0.44 Å), is the one that is dynamically favored. Also, the structure differing most from **TS1** geometry, **P3** ( RMSD = 2.5 Å), is the least formed in trajectories. Figure 11 shows how strikingly, this relationship provides an excellent correlation. The correlation between product distribution and product potential or Gibbs free energies is much worse (*R* = 0.620 and 0.617, respectively), meaning that TS and product geometries are more relevant for the trajectory outcome than product stability (Table S3). Peterson and Carpenter used geometric criteria to estimate dynamics effects on product ratios in a bifurcating reaction. They also concluded that reasonable predictions could be made on “geometric rather than energetic grounds”.^19^

**Figure 12:**
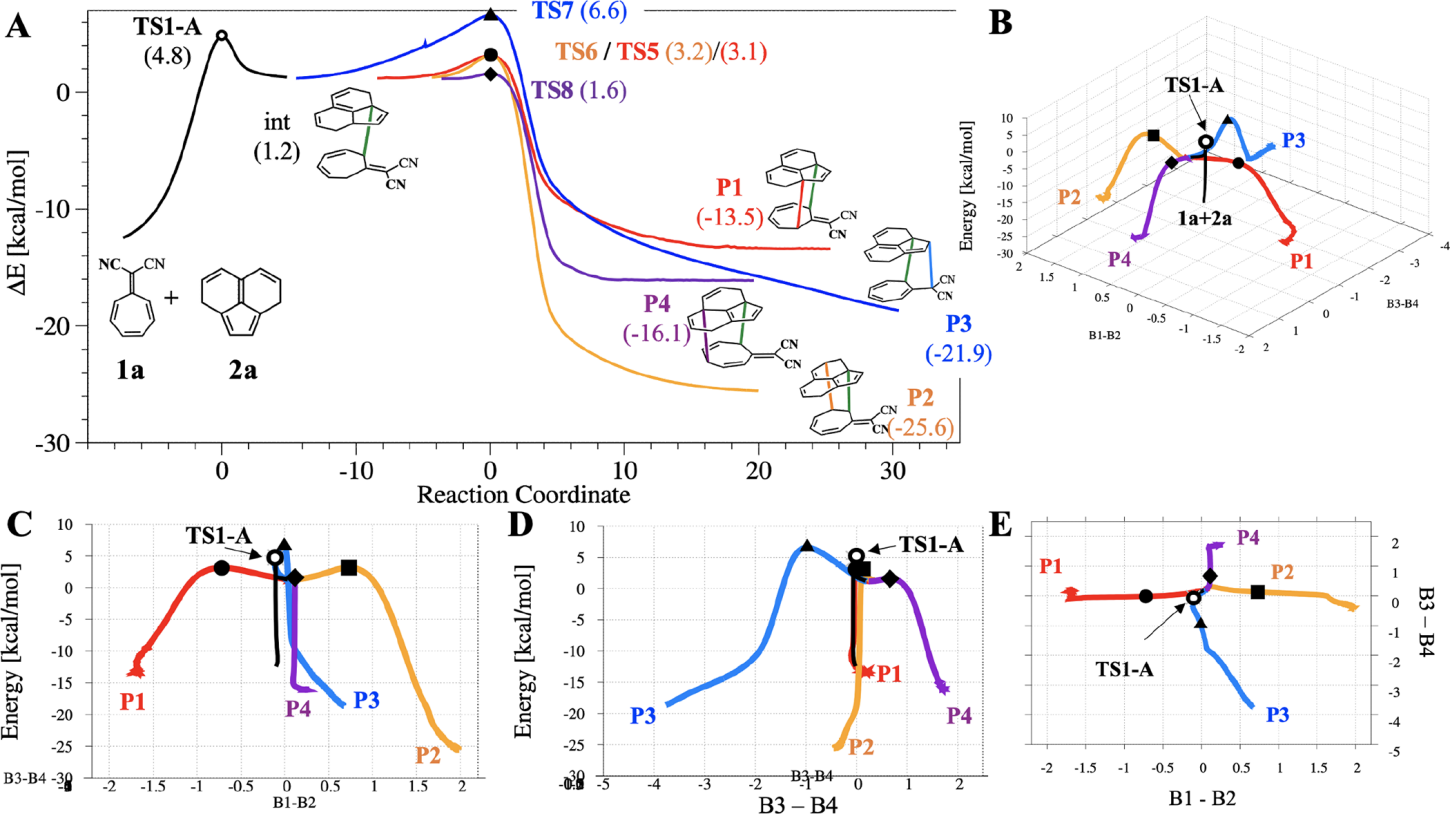
(A) IRCs for reaction A. The black curve is the IRC for **TS1-A** (not ambimodal) connecting the reactants and the intermediate. The next four IRC curves correspond to **TS7**, **TS8**, **TS9**, and **TS8**, connecting the intermediate with each of the four cycloadducts **P1**, **P2**, **P3**, and **P4**, respectively. (B–E) Different views of the same IRCs as panel A plotted on the reduced dimensionality surface obtained by plotting in the *x* axis the value of B1–B2 and in the *y* axis B3–B4 bond lengths.

By analyzing the PES for reaction A (Figure 12), stepwise and with a TS connecting the intermediate with each of the four products, we conclude that in the region around **TS1**, the slope leading to **P4** is the steepest one followed by **P2** ≈ **P1** and **P3**, which also agrees with the product distribution observed in dynamics simulations with more than half of the trajectories yielding **P4**. (the PES for reaction A and a more detailed discussion are shown in Figure S9 in the Supporting Information).

## Conclusions

We have located a heptafulvene plus vinylfulvene that leads to an ambimodal TS connecting to four products ([4 + 6]-, [2 + 8]-, [8 + 2]-, and [6 + 4]-cycloadducts) without any intervening minima. Trajectories that propagated from the ambimodal **TS1** lead to the four expected products. We found three TSs (**TS2**, **TS3**, and **TS4**) that interconvert cycloadducts. While six TSs could be present on such a potential surface, several of these have collapsed into a small number. This is likely to be a general feature of potential surfaces for higher order cycloadditions. There is a good correlation between dynamics product distribution and RMSD between the ambimodal **TS1** and product geometries.

## Computational Methods

Density functional theory computations were performed using Gaussian09^27^ and Gaussian16.^28^ Trajectory simulations were performed with the Progdyn/Gaussian interface developed by Singleton et al.^29^ All the calculations were performed at the M06-2X/6-31G(d) level of theory unless otherwise stated.

Quasiclassical trajectories were initialized in the vicinity of the TS with a normal-mode sampling method, which involves adding zero-point energy and thermal energy for each real normal mode in the TS and obtaining a Boltzmann distribution by randomly sampling a set of geometries and velocities. No additional velocities were added other than vibrations along modes perpendicular to the reaction coordinate. The trajectories were propagated forward and backward until either one of the products is formed (B0, B1, B2, B3, or B4, see labels in Figure 4, where the length is smaller than 1.5 Å) or the reactants are generated. The classical equations of motion were integrated with a velocity-Verlet algorithm, with the energies and derivatives computed “on-the-fly” by the quantum mechanical method using Gaussian09.^27^ The step length for integration is 1 fs. The time propagation was 500 fs backward and forward from the TS, for 1 ps in total.

## Supporting Information Available

Additional results as the frontier molecular orbitals for all the molecules studied, possible isomerization and dimerization competing reactions, IRC calculations from all the TS studied, IRC calculation for **TS1** computed including the solvent, and detailed IRC and comment for reaction A (PDF)
P1 representative trajectory going from the ambimodal **TS1-J** to each of the four different products (MPG)
P2 representative trajectory going from the ambimodal **TS1-J** to each of the four different products (MPG)
P3 representative trajectory going from the ambimodal **TS1-J** to each of the four different products (MPG)
P4 representative trajectory going from the ambimodal **TS1-J** to each of the four different products (MPG)

## Supplementary Material

ja2c12871_si_001.pdf

ja2c12871_si_002.mpg

ja2c12871_si_003.mpg

ja2c12871_si_004.mpg

ja2c12871_si_005.mpg
