# Supplementary material for: Computational Design of a Tetrapericyclic Cycloaddition and the Nature of Potential Energy Surfaces with Multiple Bifurcations
Source: J Am Chem Soc. 2023 Feb 9;145(7):4221–30. doi: 10.1021/jacs.2c12871 (PMC9951208; doi:10.1021/jacs.2c12871)
Supplement: Supplementary file 1 — ja2c12871_si_001.pdf [file ja2c12871_si_001.pdf]

Computational Design of a Tetrapericyclic Cycloaddition and the Nature of Potential Energy Surfaces with Multiple Bifurcations

Ana Martin-Somer[1], Xiao-Song Xue[2], Cooper S. Jamieson[2], Yike Zou[2], K.N. Houk[2]

[1]Departamento de Química, Facultad de Ciencias, Módulo 13 Universidad Autónoma de Madrid, Campus de Excelencia UAM-CSIC Cantoblanco, 28049 Madrid, Spain

[2]Department of Chemistry and Biochemistry, University of California, Los Angeles, Los Angeles, California 90095, United States

SUPPORTING INFORMATION

Table of Contents

1. ORBITALS AND ELECTRONIC STRUCTURE CALCULATIONS. 2

2. IRC CALCULATIONS....................4

3. DYNAMICS....................7

4. CORRELATION BETWEEN GEOMETRIES AND DYNAMICS PRODUCT DISTRIBUTION. 8

6. REFERENCES....................9

1. Orbitals and electronic structure calculations.

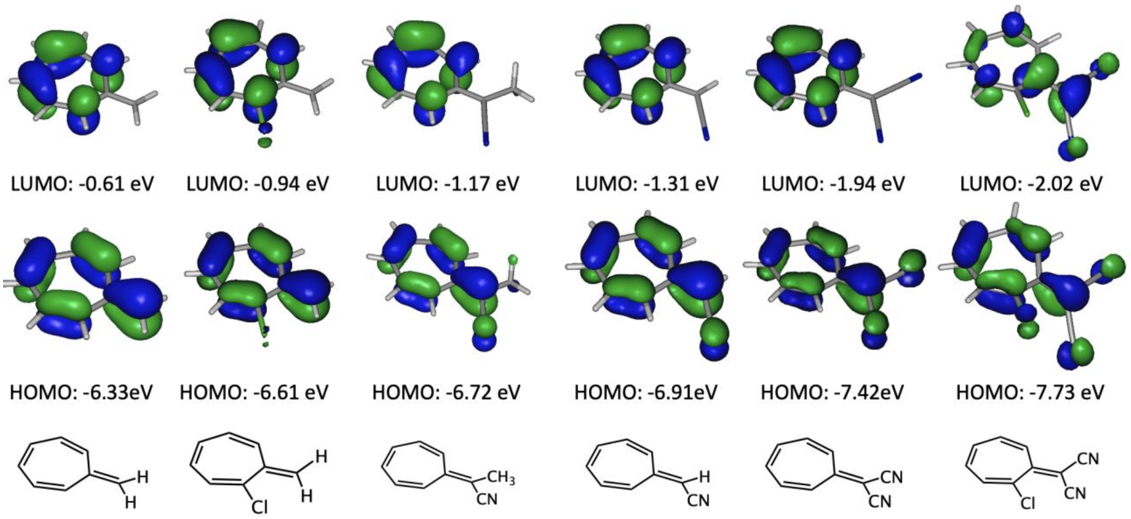

Figure S1: Frontier molecular orbitals for heptafulvene derivatives (**1**).

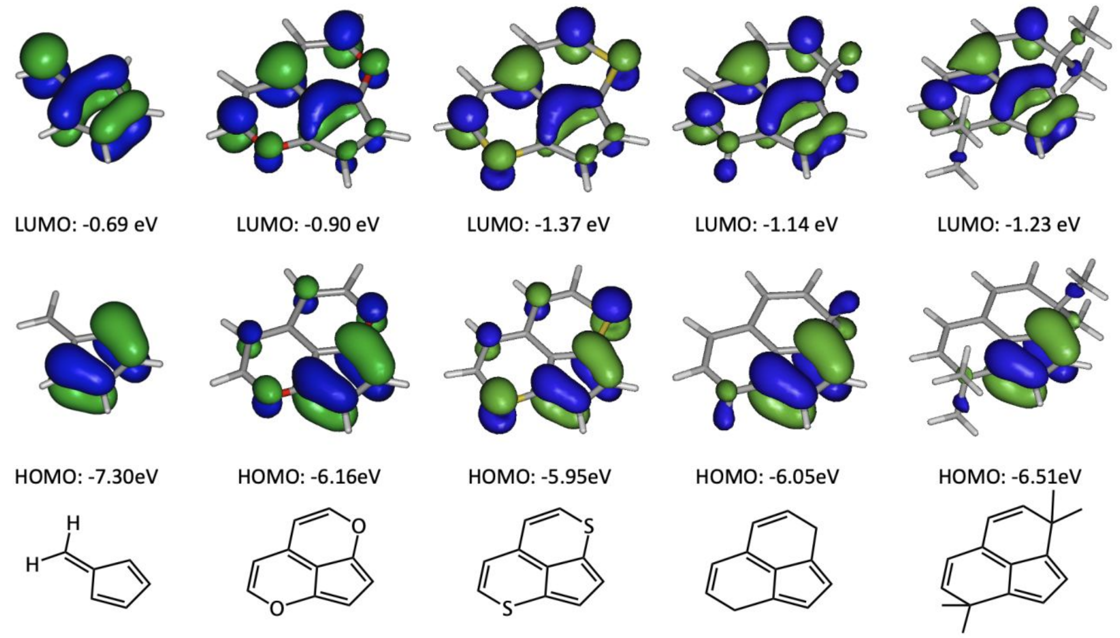

Figure S2: Frontier molecular orbitals for pentafulvene derivatives (**2**).

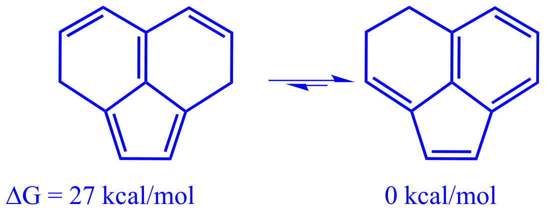

Figure S3: Isomerization reaction of the pentafulvene methylene derivative.

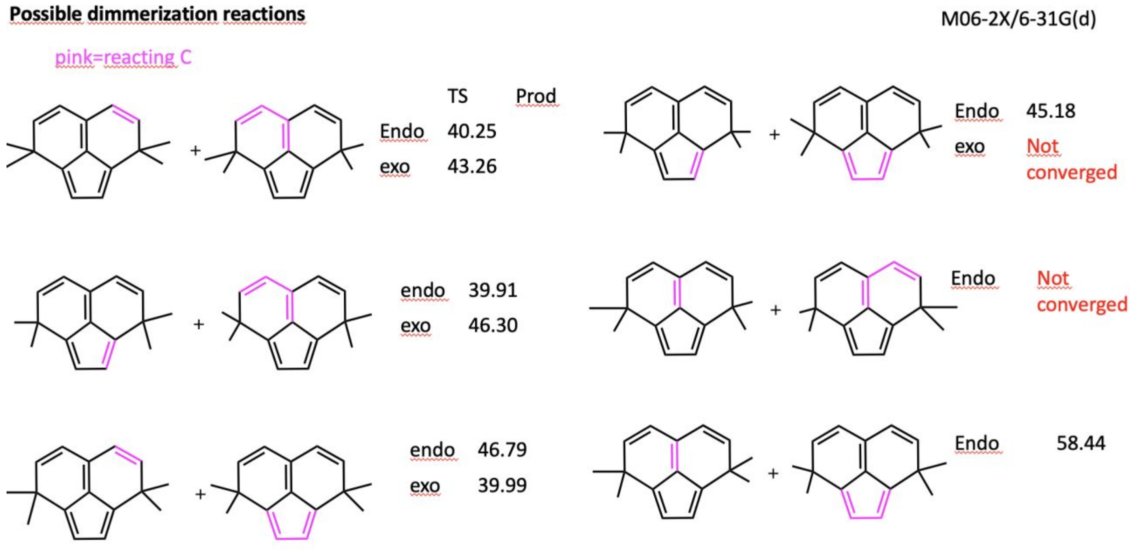

Figure S4: Competing dimerization reactions of the heptafulvene derivative

2. IRC calculations

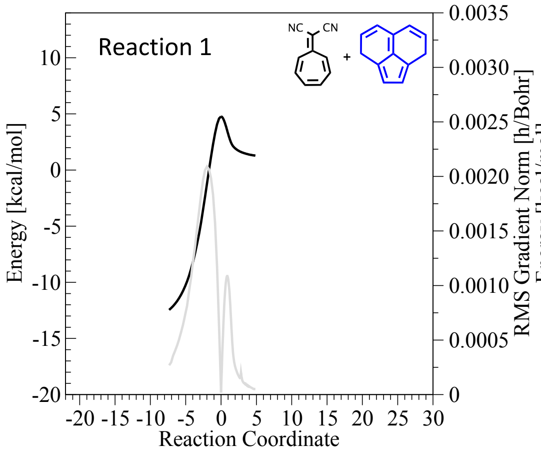

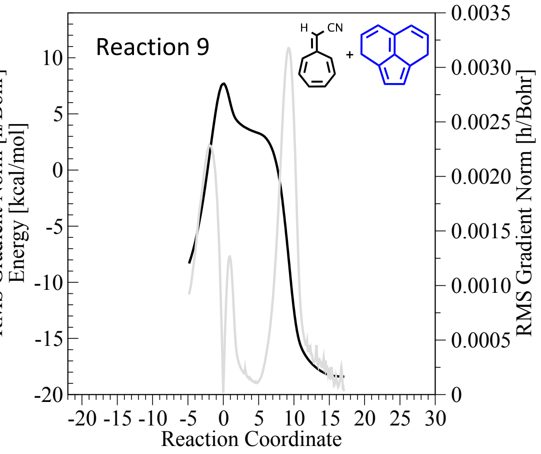

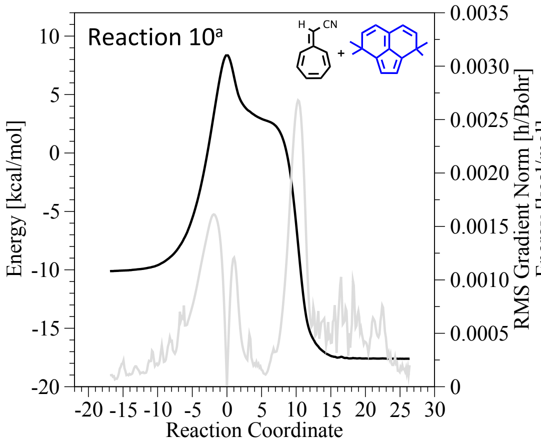

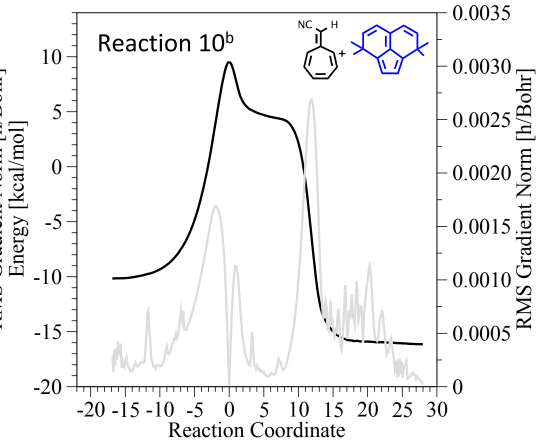

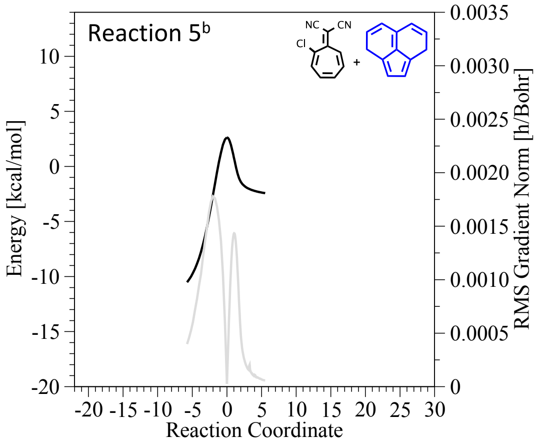

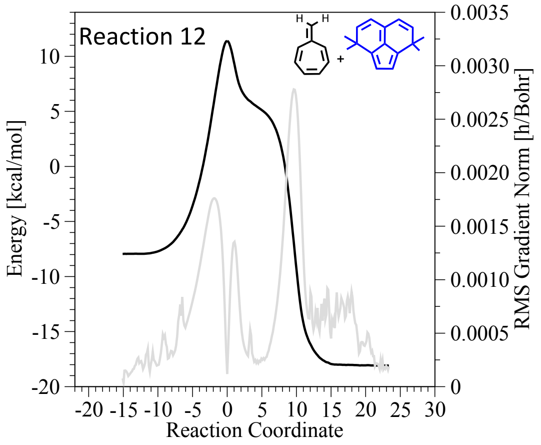

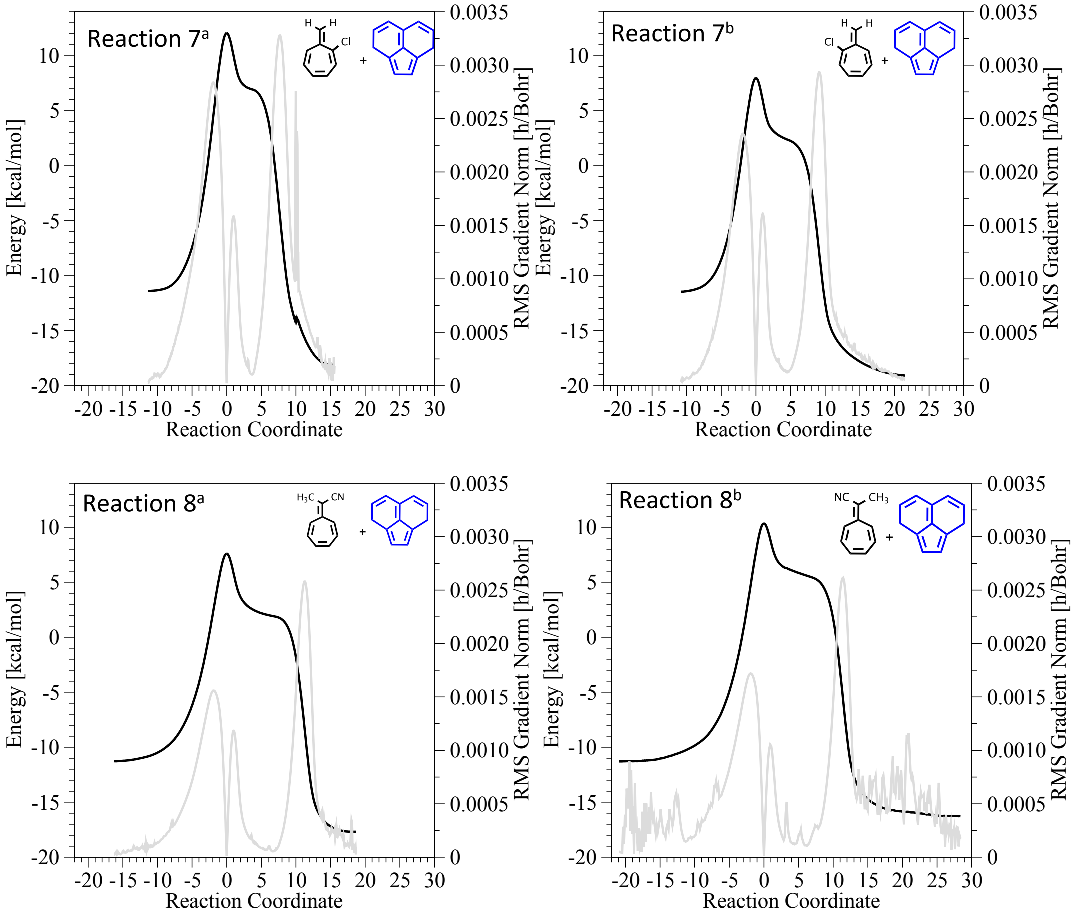

Figure S5: Intrinsic Reaction Coordinate (IRC) calculation for reactions in Table 1 of the manuscript (black curve) and gradient norm (grey line).

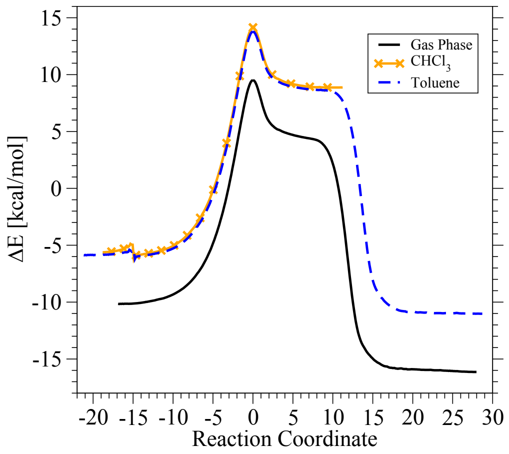

Figure S6: Intrinsic Reaction Coordinate (IRC) calculation for the ambimodal TS corresponding to reaction J^b in gas phase (black line) and with solvents $CHCl_3$ (orange cross line) and toluene (blue dashed line).

We considered the effect of the solvent (toluene and chloroform) using the SMD continuum solvation model[1] (figure S6). Since the possible intermediate would have a large dipole moment the effect of the solvent is to stabilize it. The more polar the solvent

the most stabilized gets the intermediate. Therefore, toluene (ε = 2.3741) displaces the IRC curve about 4 kcal/mol higher, while the shape keeps being the same. However, a more polar solvent like chloroform (ε = 4.7113) causes the lost of the ambimodal character of TS1 since the intermediate is stabilized.

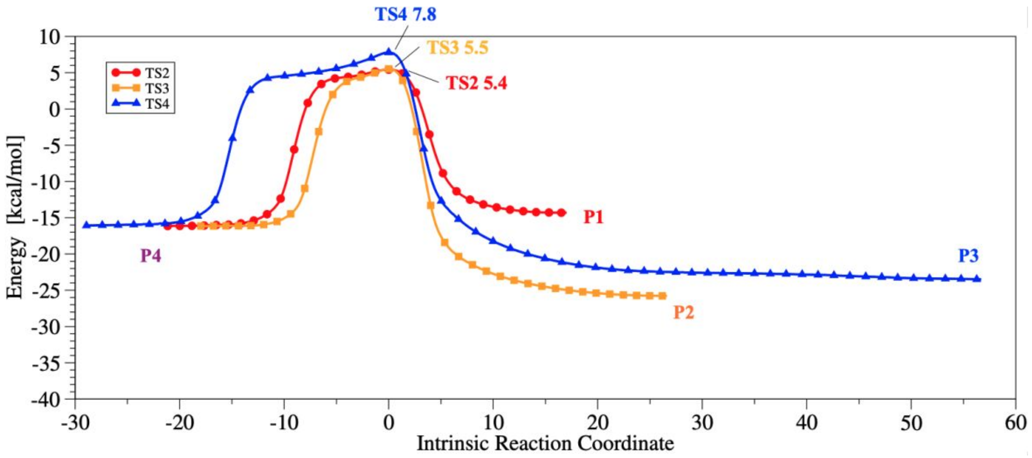

Figure S7: IRC from **TS2**, **TS3** and **TS4**.

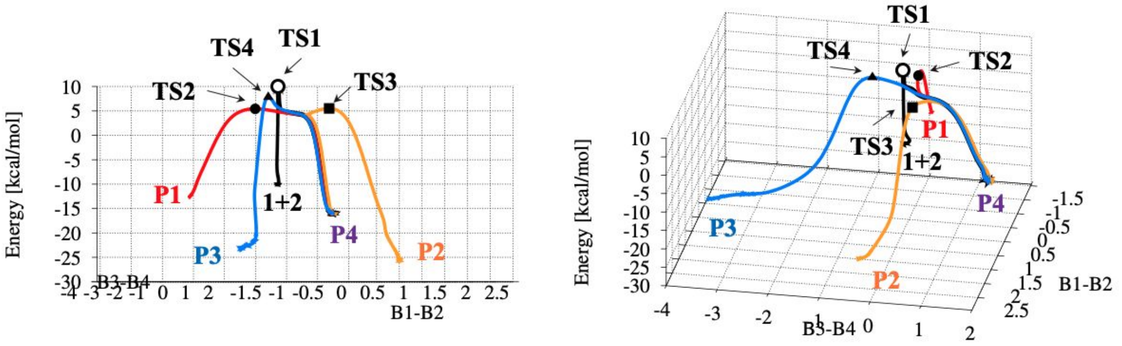

Figure S8: IRC from **TS1-4** plotted on the reduced dimensionality surface obtained by plotting in the x-axis the value of B1- B2 and in the y-axis B3 - B4 bond lengths. The empty circle, circle, triangle and square mark the coordinates for **TS1**, **TS2**, **TS3** and **TS4** respectively. Left and right are different perspectives from Figure 9B and 9C in the main text.

3. Dynamics

Table S1: Results of dynamics simulations. The numbers within parentheses correspond to the percentage over reactive trajectories. The numbers within brackets correspond to the percentage over the total number of trajectories. 5 trajectories and 4 trajectories for reaction H[a] and J[a] respectively, yielded other results.

	Reaction A	Reaction E[b]	Reaction H[a]	Reaction J[a]	Reaction J[b]
**Product 1**	4 (11)	1(4)	10 (10)	8 (9)	26 (17)
**Product 2**	9 (25)	10 (42)	24 (23)	11 (13)	17 (11)
**Product 3**	0	0	0	0	**4 (3)**
**Product 4**	23(64)	13 (54)	70 (67)	69 (78)	105 (69)
**Total reactives**	36 [40]	24 [24]	104 [91]	88 [94]	152 [93]
**Recrossing**	12 [13]	3 [3]	3 [3]	1 [1]	5 [3]
**Intermediate**	43 [47]	73 [73]	2 [2]	1 [1]	7 [4]
**Total trajectories**	91	100	114	94	164

Table S2: Dynamics results for product interconversion.

	**TS 2**	**TS 3**	**TS 4**
**P1->P4**	49 (78)	1 (2)	--
**P2->P4**	4 (6)	45 (75)	--
**P3->P4**	--	--	39 (67)
**P1->P2**	3 (5)	3 (5)	--
**P1->P3**	--	--	8 (14)
**P2->P3**	--	--	5 (9)
**P1->P1**	2 (3)	--	--
**P2->P2**	1 (2)	1 (2)	--
**P3->P3**	--	--	2 (3)
**P4->P4**	2 (3)	5 (8)	1 (2)
**Int->P1**	1 (2)	--	--
**Int->P2**	--	5 (8)	--
**Int -> P4**	1 (2)	--	--
**Reactants-> P3**	--	--	4 (7)
**TOTAL**	63	60	58

4. Correlation between geometries and dynamics product distribution.

Table S3: RMSD, ΔG, ΔE, partial bond lengths at the TS and product distribution from dynamics for reaction 10[b]. The last row is the correlation coefficient of each column with the % of trajectories.

	Bond length @TS	RMSD	ΔE	ΔG	% trajectories
**Prod1**	2.91	1.88	-14.30	6.09	17
**Prod2**	2.97	1.80	-25.79	-6.52	11
**Prod3**	2.98	2.41	-27.84	-9.38	3
**Prod4**	3.11	0.44	-16.15	3.93	69
**R^2**	0.850	-0.981	0.620	0.617	

Comparison with reaction A

Finally, in other to gain more insight into the reaction PES, we used reaction A, with an intermediate between **TS1** and products, and therefore not ambimodal. The reactants are different, but the reaction can be compared since the π reacting core is the same.

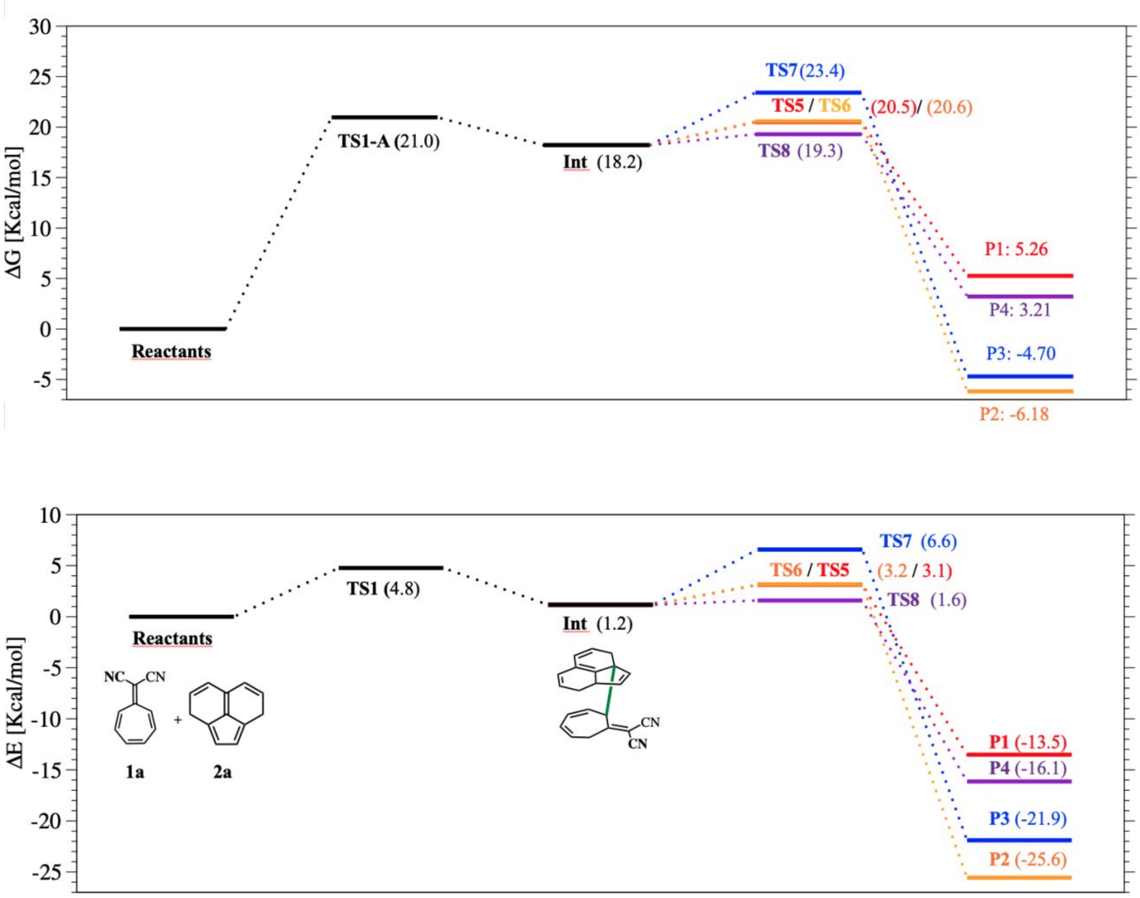

Figure S9: (**A**) Gibbs free energy and (**B**) potential energy diagram for reaction A.

Figure S10 show the Gibbs free energy (A) and potential energy (B) diagrams for reaction A. Since trajectories move in the PES, we though more convenient to use the PES for the discussion. The trends are the same than in the Gibbs free energy diagram. After **TS1**, there is an intermediate, where B0 bond has already been formed. This intermediate is 3.6kcal/mol more stable than **TS1**. From the intermediate we have four TS connecting it with the four products (**TS5**, **TS6**, **TS7**, and **TS8**). The highest in energy is **TS7** (6.6 kcal/mol) less stable than **TS1** (4.8kcal/mol). Since trajectories are started at **TS1**, with the energy of this TS, it is normal that no trajectory yields **P3** since the reactants do not have energy enough to go through this barrier. Second higher in energy are **TS5** and **TS6** with almost the same energy (3.1 and 3.2 respectively). Trajectories leading to **P2** are more than double than trajectories yielding **P1**, 25% vs 11%, probably due to the fact that **P2** is much more stable than **P1**. **TS8** has the lowest energy (1.6kcal/mol), only 0.4 kcal/mol above the intermediate and therefore, **P4** is the main product from trajectories. Though in reaction J^b there is no intermediate, and therefore **TS5**, **TS6**, **TS7**, and **TS8** do not exist, these results can be extrapolated to explain its reactivity. Around **TS1** the PES will be flatter in the region leading to **P3** than in the regions leading to **P2-P3** and **P4**. Trajectories will follow preferentially the steepest slope which would be the path leading to **P4**.

6. References

1. A. V. Marenich, C. J. Cramer, D. G. Truhlar, *J. Phys. Chem. B* **2009**, 113, 6378-6396.
